# Additive Value of Pyridostigmine to Silodosin in the Management of Acute Urinary Retention Secondary to Benign Prostatic Hyperplasia: A Randomized Controlled Trial

**DOI:** 10.3390/jcm14030674

**Published:** 2025-01-21

**Authors:** Ahmed G. Mohamed, Hany F. Badawy, Amira S. A. Said, Mohammad M. Al-Ahmad, Al Shaimaa Ibrahim Rabie, Hager Salah, Ramy Massoud, Raghad R. S. Hussein, Doaa Mahmoud Khalil, Ahmed Yousef, Rabie M. Ibrahim

**Affiliations:** 1 Department of Urology, Faculty of Medicine, Beni-Suef University, Beni Suef 62521, Egypt; dr.ahmed.gamal@med.bsu.edu.eg (A.G.M.); hanyfathy86@gmail.com (H.F.B.); ahmed0122@med.bsu.edu.eg (A.Y.); rabee75@yahoo.com (R.M.I.); 2 Department of Clinical Pharmacy, Faculty of Pharmacy, Beni-Suef University, Beni-Suef 62521, Egypt; amira.ahmed@aau.ac.ae; 3 Department of Clinical Pharmacy, College of Pharmacy, Al Ain University, Al Ain P.O. Box 64141, United Arab Emirates; mohammad.alahmad@aau.ac.ae; 4 Clinical Nutrition Department, Health Insurance Authority, Faiyum 63511, Egypt; 5 Pharmaceutical Services Kingdom Bahrain, Busaiteen 24343, Bahrain; hager.salah@rocketmail.com; 6 Department of Urology, Faculty of Medicine, MUST University, Giza 12566, Egypt; ramy.masoud@must.edu.eg; 7 Public Health and Community Medicine Department, Faculty of Medicine, Beni-Suef University, Beni-Suef 62514, Egypt; doaamahmoud@med.bsu.edu.eg

**Keywords:** acute urinary retention, BPH, pyridostigmine bromide, silodosin, TWOC

## Abstract

**Background**: In men with progressive benign prostatic hyperplasia (BPH), acute urine retention (AUR) stands out as one of the most severe outcomes associated with aging. AUR is characterized by a sudden, painful inability to urinate. This research investigates the potential benefits of adding pyridostigmine to silodosin in the management of acute urinary retention secondary to benign prostatic hyperplasia. **Methods**: Patients aged 50 and above experiencing their first episode of AUR due to BPH, with a retention volume below 1000 milliliters, were enrolled in this study. A total of 140 patients were randomized into two groups: Group A received a daily dose of pyridostigmine bromide (60 mg tablet) alongside an 8 mg silodosin capsule, while Group B received a daily dose of silodosin (8 mg capsule) only. Trial registration number: NCT06319469 13 March 2024. **Results**: Among the 140 patients, 58 (82.9%) in Group A successfully underwent a trial without catheter (TWOC), compared to 47 (67.1%) in Group B. Group A exhibited significant improvements in international prostatic symptom score (IPSS) and uroflowmetry (UFR) at both 2 weeks and 3 months, with *p*-values of 0.001 and 0.003, respectively. Regarding postvoid residual volume (PVR), both groups were initially matched at baseline, showing significant continuous improvement at the second week and third month. **Conclusions**: The combination of pyridostigmine bromide (60 mg tablet) with silodosin proved to be more effective than silodosin alone in managing acute urinary retention caused by BPH. This was particularly true for patients who were suspected to have detrusor underactivity in addition to BPH.

## 1. Introduction

Acute urine retention (AUR) is often seen as the most severe outcome for aging men with progressive benign prostatic hyperplasia (BPH). It is characterized by a sudden and painful inability to urinate freely [1].

Every year, between 0.44% and 25% of men seen in routine urological care experience AUR [2]. A notable US cohort study found that a 60-year-old man has a 23% chance of experiencing an AUR episode if he lives for another 20 years [3].

The typical approach to managing AUR involves immediate bladder catheterization, followed by catheter removal to check if normal urination resumes, known as a trial without catheter (TWOC) [4].

AUR can emanate from the following conditions:

1. Bladder outlet obstruction (BOO) caused by one or more factors.

2. A weak detrusor, resulting from one or more etiologies, manifesting as weak bladder contractions, potentially due to compromised sensory or motor innervation of the detrusor muscle [5].

After the insertion of a bladder catheter, further therapeutic interventions might include prolonged catheterization or surgical interventions for BPH. The elevated risks of bleeding, infection, sexual dysfunction, incontinence, and recurrence associated with immediate surgery for AUR—or potential adverse outcomes like bacteriuria, fever, and urosepsis from extended catheterization—underscore the preference for TWOCs [6].

Generally, an alpha-blocker is given before starting TWOC, significantly boosting the chances of success, especially when AUR is due to heightened sympathetic activity in the prostatic smooth muscles [7].

Chronic BOO can lead to detrusor underactivity (DU), resulting in detrusor hypertrophy. This condition significantly impairs bladder voiding efficiency in men with BOO [8].

Yamanishi et al. have shown that combining cholinomimetics with alpha blockers is more effective than using monotherapy for treating patients with decreased bladder activity [9].

Chronic bladder overexpansion leads to damage in the postsynaptic parasympathetic ganglia and the destruction of muscle fibers, resulting in detrusor muscle weakness and impairment of detrusor contractility. Lee et al. reported that 37% to 47% of patients with benign prostatic hyperplasia exhibited decreased detrusor contractility. For all these reasons, this study aimed to evaluate the efficacy of combining pyridostigmine bromide (a parasympathomimetic) with alpha-blockers in treating AUR [10].

## 2. Materials and Methods

The study was conducted in accordance with the declaration of Helsinki guidelines and approved by the Institutional Review Board Statement of the Faculty of Medicine, Beni Suef University, under the permission number FMBSUREC/06122022/Mohamed (6 December 2022).

A parallel-group randomized controlled trial (simple randomization using Microsoft Excel) was conducted in patients attending the urology clinic at the Faculty of Medicine, Beni Suef University. Excel’s built-in random number generation functions were used to create the allocation sequences. To ensure a clear and reproducible randomization process, we used Excel’s built-in random number generation function (RAND()) to assign participants to either Group A or Group B. Generation of Random Numbers: A random number was generated for each participant using the RAND() function in Microsoft Excel. The random numbers generated were then sorted in ascending order.

Assignment to Groups: Participants corresponding to the top 50% of the sorted random numbers were assigned to Group A, while those corresponding to the bottom 50% were assigned to Group B. This approach ensured equal probability of allocation to either group while maintaining simplicity and reproducibility. 

The study included patients over 50 years old experiencing their first episode of AUR due to BPH, having a retention volume of less than 1000 mL. Researchers recorded clinical data and demographic traits, including medical histories of diabetes and hypertension, retention volume, prostate-specific antigen levels, and prostate size assessed via abdominal and pelvic ultrasounds.

Exclusion criteria included patients with urinary tract infections, ongoing BPH treatment, recurrent urine retention, unsuccessful prior voiding trials, retention volumes over one liter, history of prostatic or bladder neck surgery, diagnoses of prostate carcinoma, urethral stricture, urinary bladder stones, neurogenic bladder, renal failure, or liver disease.

After fixation of the urinary catheter, the principal investigator randomized 140 patients into two groups: Group A received an 8 mg silodosin capsule once daily and a 60 mg pyridostigmine bromide tablet 3 times daily, while Group B received an 8 mg silodosin capsule once daily alone. The drugs were administered over one week, followed by TWOC. Patients successfully completed the TWOC if they voided at least 100 mL of urine and had a post void residual (PVR) volume under 100 mL. Post-TWOC, both groups received a single 8 mg dose of silodosin; pyridostigmine was discontinued.

This study adheres to CONSORT guidelines for clinical trial reporting. Patients were evaluated using international prostatic symptom score (IPSS), PVR volume, and uroflowmetry (UFR). Those experiencing AUR with significant pain or obstructive lower urinary tract symptoms (LUTSs), requiring catheterization and scheduled transurethral resection of the prostate, were considered unsuccessful in the watchful waiting trial. Successful TWOC patients were re-evaluated at two weeks and three months, with measurements taken for UFR, PVR volume, and IPSS.

## 3. Results

### 3.1. Sample Size Calculation

**Figure 1:**
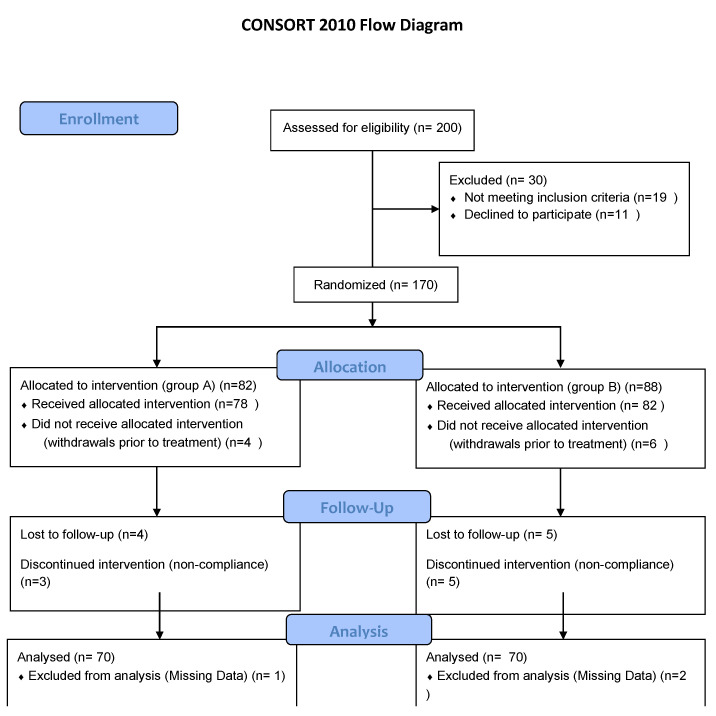
Flow diagram of the study.

Based on the sample size calculation using G. power 3.1 for Windows, a two-tailed *t*-test was employed to detect a difference between two independent means of residual urine with an effect size of 0.7. With an alpha error probability of 0.05 and a desired power of 0.95, the required sample size was determined to be 55 participants per group, totaling 110 participants. Accounting for a potential 20% dropout rate in each group, the adjusted sample size should be 70 participants per group, leading to a total of 138 participants for the entire study, as shown in Figure 1.

### 3.2. Statistical Analysis

Data analysis was conducted using SPSS version 27. Due to non-normal distribution, scale data were presented as the median and interquartile range. Quantitative data were provided as numerical values and proportions, indicating categorical factors. The Mann–Whitney U test was used to compare groups based on scale data, while the Wilcoxon signed rank test was used for subsequent analysis within each group. Associations between groups for categorical data were assessed using the chi-square test. A multivariable binary logistic regression analysis was performed to identify variables influencing the success of TWOC with pyridostigmine. A *p*-value below 0.05 was considered statistically significant.

In this study, there was no significant difference between the studied groups regarding age, diabetes and hypertension distribution, PSA, and volume at catheterization (*p* value > 0.05). However, the prostate volume was significantly larger in the silodosin and pyridostigmine group, with a median (IQR) of 57.00 (47.75, 75.00) grams, compared to 48.50 (40.00, 65.00) grams in the other group, as indicated in (Table 1).

On the 7th day, the primary endpoint of the study showed that 58 (82.9%) patients in the silodosin and pyridostigmine group achieved a successful TWOC, compared to 47 (67.1%) in the silodosin group. Meanwhile, 12 (17.1%) patients in both groups required re-catheterization. The study demonstrated a statistically significant difference between the groups, with 23 (32.9%) patients in both groups needing re-catheterization, as shown in Table 2.

For the secondary outcomes, we followed up on successful cases at 2 and 3 months for IPSS score, UFR, and PVR. Significant improvements in IPSS scores were observed between the 2nd week and the 3rd month, with greater improvements in the silodosin and pyridostigmine group [median (IQR), 11.00 (11.00, 12.00)] compared to the silodosin-only group [median (IQR), 13.00 (12.00, 13.00)], with a *p* value < 0.001, as illustrated in Table 3.

Uroflowmetry results were also significantly better in the silodosin and pyridostigmine group compared to the silodosin-only group at the 2nd week and 3rd month, with improvements in both groups over this period.

Regarding post void residual urine, both groups showed significant continuous improvement at the 2nd week and 3rd month. There was no significant difference between the groups at the 2nd week. However, by the 3rd month, residual urine was significantly lower in the silodosin and pyridostigmine group [median (IQR), 40.00 (30.00, 50.00)] than in the silodosin-only group [median (IQR), 50.00 (40.00, 50.00)] (Table 3).

After adjusting for baseline characteristics, the combination of pyridostigmine and silodosin was found to increase the probability of successful TWOC to 2.867 (95% CI, 1.143, 7.193) compared with silodosin alone. However, neither diabetes nor increased volume at catheterization contributed to an increased probability of success, as represented in Table 4.

## 4. Discussion

BPH is a progressive, degenerative condition marked by the gradual worsening of symptoms over time. It can lead to severe consequences, such as acute urinary retention, which may require surgical interventions associated with BPH in some individuals [11]. 

Acute urinary retention constitutes a prevalent urological emergency, defined by the inability to void urine, typically accompanied by intensifying pain and distress [12]. Diagnosis is usually clinically apparent, supported by a characteristic patient history and palpation or percussion revealing an enlarged bladder. Ultrasound may confirm the diagnosis. Initial patient management typically occurs in primary care or hospital emergency departments (EDs) via urethral catheterization [13].

TWOC is usually advocated after two to seven days; however, the optimal timing remains undetermined [14].

Observational studies indicate that the success rates for TWOCs range between 23% and 28% [15].

A systematic review and meta-analysis by D. Guang-Jun et al. evaluated the effectiveness of alpha-blockers in treating acute urine retention caused by BPH. Their findings showed that 56.8% of patients using alpha-blockers experienced a successful TWOC, compared to 38.9% in the control groups [16].

Based on a systematic analysis completed in 2014, encompassing nine randomized studies, the administration of alpha-1-adrenergic antagonists before the extraction of urethral catheters for AUR was determined to possess a reasonable level of evidence. According to the research, the antagonists examined in the study were shown to be correlated with a higher rate of success in trials conducted without the use of a catheter. The relative risk was calculated to be 1.55, with a 95% confidence interval ranging from 1.36 to 1.76. Furthermore, it was observed that the occurrence of negative consequences was minimal [17].

Currently, the standard treatment for men with AUR is TWOC following alpha-blocker therapy [18]. 

Association between benign prostatic obstruction and impaired detrusor contractility is not uncommon as documented by Coolsaet and Blok, where they established that 78% of obstructed men had decreased contraction power [19].

Previous studies have shown evidence that the simultaneous administration of a cholinergic agent and an alpha-blocker results in greater effectiveness than the separate utilization of these agents. The participants in the study conducted by Yamanishi et al. were classified into three discrete categories. The participants assigned to group 1 were given cholinomimetic drugs, namely bethanechol chloride at a daily dose of 60 mg or distigmine bromide at a daily dose of 15 mg. In contrast, the individuals comprising group 2 were administered urapidil, an alpha-blocker, at a daily dosage of 60 mg. The participants allocated to group 3 were administered a pharmaceutical regimen of cholinomimetics in conjunction with alpha-blockers. After four weeks, significant decreases in IPSS and PVR volume were observed in both the groups receiving alpha-blocker treatment and combination therapy [9].

However, the effects of cholinomimetics on weak detrusor contractility associated with BOO in the context of treating AUR with TWOC have not been previously studied. This study utilized a combination of pyridostigmine and silodosin, revealing a significant increase in TWOC success rates to 82.9%. In contrast, the TWOC success rate with silodosin alone was 67.1%, a statistically significant difference (*p* value = 0.032). Moreover, the efficacy of TWOC in the group receiving pyridostigmine with silodosin (82.9%) surpassed that reported in previous studies on TWOC using silodosin conducted by Patil et al. (60%) [20] and Ginka et al. (72.33%) [21]. Additionally, the success rate of TWOC with pyridostigmine and silodosin was higher than the success rates reported for tamsulosin in studies by Hua et al. (61%) [22], Lucas et al. (45.3%) [23], and Agrawal et al. (70%) [24].

The increase in PVR volume at the beginning of the study and in the second week showed no significant difference between the two groups. However, after three months, there was a statistically significant improvement in the pyridostigmine with silodosin group compared to the other group, with a *p*-value of less than 0.001. Furthermore, statistical significance was observed in both groups at the second week and the three-month mark, with a *p*-value of less than 0.001.

In contrast to the odds reported by Kumar et al., the present investigation observed that patients in the group receiving pyridostigmine in combination with silodosin had a greater likelihood of achieving effective voiding after undergoing a trial without a catheter (TWOC). The study findings revealed that the likelihood ratio was calculated to be 2.867, with a 95% confidence interval ranging from 1.143 to 7.193. Based on the study’s findings, it was determined with a 95% confidence interval ranging from 1.26 to 4.38 that the likelihood of successful voiding after a trial without a catheter (TWOC) was 2.35 times greater in the silodosin group compared to the placebo group, according to Kumar et al. [18].

In the context of multivariate analysis, our findings indicate a positive correlation between grouping and the success rate of TWOC. Conversely, we observed negative associations between the success of TWOC and DM and greater retention volume. Nevertheless, it has been observed that neither age nor prostate volume had any significant influence on the efficacy of TWOC procedures. This finding aligns with the results published by Kumer et al. [18] and McNeil et al. [25].

Based on our research findings, both groups demonstrated comparable outcomes regarding the observed enhancements in IPSS scores during the second week. The pyridostigmine + silodosin group demonstrated a much higher statistical significance during the three months, as indicated by a *p*-value of less than 0.001. Moreover, in both cohorts, the statistical significance of the enhancement in the IPSS was more pronounced at the 3-month mark compared to the second week. These results were comparable with the study conducted by Sugaya et al. where they found that IPSS score and PVR decreased significantly in the group receiving a combination of an alpha-blocker and a daily dosage of distigmine (5 mg) [26].

At the start of the study and at the second week, there was no significant difference in PVR volume between the two groups. However, by the three months, the pyridostigmine and silodosin group showed a statistically significant improvement compared to the other groups, with a *p*-value of less than 0.001. Both groups also showed significant improvements at the second week and three-month follow-ups, with *p*-values of less than 0.001.

UFR is one of the studied parameters in our study. We found that UFR values in the group receiving pyridostigmine in combination with silodosin were statistically significantly higher at the 2nd week and 3-month follow ups than in the silodosin-only group, with a *p* value of 0.003 *, <0.001 *, respectively.

Two limitations of our study include the small sample size and the inability to use urodynamics to identify cases of underactive bladders. Nonetheless, this research is groundbreaking as it explores the combined use of pyridostigmine and silodosin to manage acute urinary retention caused by benign prostatic hyperplasia (BPH), and we recommend extending our research to a multicenter study using urodynamic analysis in a large number of patients with long-term follow up.

## 5. Conclusions

In conclusion, combining pyridostigmine bromide (60 mg tablets) with silodosin proved more effective than using silodosin alone in treating acute urinary retention caused by benign prostatic hyperplasia. This was especially true for cases where detrusor weakness was present alongside BPH.

## Figures and Tables

**Table 1:** Baseline characteristics of the studied groups.

Items	Silodosin and Pyridostigmine (No = 70)	Silodosin Only (No = 70)	*p* Value
Age (median [IQR])	62.00 (59.00, 65.25)	64.00 (59.75, 67.00)	0.206
HTN	18 (25.7%)	17 (24.3%)	0.845
DM	14 (20.0%)	16 (22.9%)	0.680
PSA (median [IQR])	2.30 (1.90, 2.65)	2.25 (1.95, 2.50)	0.433
Prostate volume (median [IQR])	57.00 (47.75, 75.00)	48.50 (40.00, 65.00)	0.046 *
Volume at catheterization (median [IQR])	740.00 (665.00, 800.00)	740.00 (680.00, 750.00)	0.978

IQR: Interquartile range. * *p* value is significant. DM: diabetes mellitus; HTN: hypertension; PSA: prostate-specific antigen.

**Table 2:** Outcomes of the study (success) after one week.

**Items** **Outcome**	**Silodosin and Pyridostigmine** **(No = 70)**	**Silodosin Only** **(No = 70)**	***p* Value**
Failed	12 (17.1%)	23 (32.9%)	0.032 *
Succeeded	58 (82.9%)	47 (67.1%)	

* *p* value is significant.

**Table 3:** Comparison between the studied groups regarding the IPSS score and uroflowmetry (successful cases only).

Items	Silodosin and Pyridostigmine (No = 58) (Median [IQR])	Silodosin Only (No = 47) (Median [IQR])	*p* Value Between Groups
IPSS score at 2nd week	18.00 (17.00, 19.00)	18.00 (17.00, 19.00)	0.861
IPSS score at 3 months	11.00 (11.00, 12.00)	13.00 (12.00, 13.00)	<0.001 *
*p* value at 2 weeks vs. 3 months	<0.001 *	<0.001 *	
Uroflowmetry at 2nd week	13.00 (12.00, 14.00)	12.00 (11.00, 13.00)	0.003 *
Uroflowmetry at 3 months	15.00 (14.00, 16.00)	14.00 (13.00, 14.00)	<0.001 *
*p* value at 2 weeks vs. 3 months	<0.001 *	<0.001 *	
Baseline post voiding residual urine	70.00 (67.50, 80.00)	70.00 (60.00, 80.00)	0.496
Post voiding residual urine at 2nd week	60.00 (50.00, 70.00)	60.00 (50.00, 70.00)	0.457
Post voiding residual urine at 3 months	40.00 (30.00, 50.00)	50.00 (40.00, 50.00)	<0.001 *
*p* value			
Baseline vs. 2 weeks	<0.001 *	<0.001 *	
2 weeks vs. 3 months	<0.001 *	<0.001 *	
Baseline vs. 3 months	<0.001 *	<0.001 *	

* *p* value is significant. IQR: interquartile range. IPSS: International prostatic symptoms score.

**Table 4:** Binary logistic regression analysis for the prediction of successful catheter removal after adjustment for other baseline characteristics.

Independent Variables	*p* Value	OR	95% CI for OR	
			Lower	Upper
Silodosin and pyridostigmine	0.025	2.867	1.143	7.193
Age/years	0.432	0.970	0.899	1.046
DM	0.035	0.318	0.110	0.921
HTN	0.484	0.695	0.252	1.922
Baseline prostate volume	0.408	0.992	0.973	1.011
Volume at catheterization	0.002	0.989	0.982	0.996

OR = odds ratio. CI: confidence interval.

## Data Availability

The data will be made available by the corresponding author upon request.

## Abbreviations

AUR	Acute urine retention
BPH	benign prostatic hyperplasia
TWOC	trail without catheter
IPSS	international prostatic symptom score
PVR	post-void residual volume
BOO	bladder outlet obstruction
DU	Detrusor underactivity
UFR	uroflowmetry
LUTS	lower urinary tract symptoms
